# Complex oscillations in the Belousov–Zhabotinsky batch reaction with methylmalonic acid and manganese(ii)

**DOI:** 10.1039/d1ra01734a

**Published:** 2021-05-04

**Authors:** Glen A. Frerichs, Desmond Yengi

**Affiliations:** Department of Chemistry, Westminster College 501 Westminster Ave Fulton MO 65251 USA glen.frerichs@westminster-mo.edu +1-573-310-4108; College of Arts and Sciences, Ohio Valley University 1 Campus View Drive Vienna WV 26105 USA

## Abstract

The Belousov–Zhabotinsky (BZ) oscillating chemical reaction involves the oxidation of an organic compound by the bromate ion in the presence of a metal ion catalyst such as cerium(iv), manganese(ii), or ferroin. Simple periodic oscillations are generally obtained for the BZ reaction in a batch (closed) system. However, complex oscillations have been observed for the BZ reaction in batch with malonic acid and either cerium or ferroin ions as the catalyst. We report here that fascinating complex oscillations in the potential of a Pt electrode have been found in the batch BZ reaction with methylmalonic acid (MeMA) and manganese(ii). Relatively high initial concentrations of NaBrO_3_ and MeMA are required, and the [NaBrO_3_]_0_/[MeMA]_0_ ratio is the main factor determining the type of oscillations obtained. Other relevant factors are [NaBr]_0_, [MnSO_4_]_0_, [H_2_SO_4_]_0_ or [NaOH]_0_, temperature, and stirring rate. Complex phenomena observed include mixed mode oscillations, birhythmicity, quasiperiodicity, bursting, and possible chaos. A mechanism is proposed involving the reversible formation of a manganese(iii) complex with bromomethylmalonic acid followed by two-electron oxidation to methyltartronic acid and Mn^2+^.

## Abstract 2

Mixed mode and bursting oscillations are among the complex oscillations observed in the Belousov–Zhabotinsky reaction with methylmalonic acid and Mn(ii).

## Graphical abstract

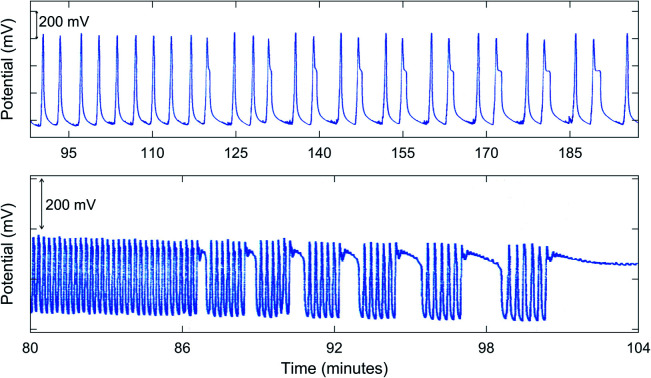

## Introduction

The oscillation patterns exhibited in the rhythmic beating of cardiac pacemaker cells, the central pattern generators that control rhythmic actions, and the bursting patterns found in various types of neurons due to changing membrane potential are similar to those observed under certain conditions in the Belousov–Zhabotinsky (BZ) reaction system. In the well-studied BZ oscillating chemical reaction an organic compound is oxidized by the bromate ion in the presence of a metal ion catalyst such as cerium(iv), manganese(ii), ruthenium(ii), or ferroin. The classical BZ system consists of malonic acid (MA), BrO_3_^−^, and Ce(iv) in H_2_SO_4_.^1^ Chemical oscillations have been observed for the BZ reaction both in a closed (batch) reactor and in a continuous-flow stirred tank reactor (CSTR). Among the organic substrates found to give oscillations with the BZ system is methylmalonic acid (MeMA).

Most commonly, the BZ reaction in batch gives simple periodic oscillations in absorbance at a fixed wavelength and/or in potential of a Pt or specific-ion electrode *versus* a reference electrode. However, in a few BZ systems with MA in batch, complex oscillations have been observed. Ruoff^2^ reported chaotic behavior in the BZ reaction catalyzed by cerium ion. Wang, Sorensen and Hynne^3^ found period doubling, intermittency, mixed mode and quasiperiodic oscillations in the BZ reaction also with cerium ion. Kawczinski *et al.*^4,5^ obtained mixed mode oscillations in the BZ reaction catalyzed by ferroin. Johnson, Scott and Thompson^6^ confirmed the complex oscillations of Wang *et al.* and modeled this behavior in the cerium-catalyzed BZ reaction. Experimental and numerical evidence for complex oscillations in the BZ reaction catalyzed by cerium in batch was reported subsequently by Kolar-Anic *et al.*^7^ and Blagojevic *et al.*^8^

Manganese(ii)-catalyzed BZ systems have been less studied than those catalyzed by cerium(iv) or ferroin. Hansen and Ruoff^9^ used NMR to observe oscillations in a closed BZ system using MeMA and catalyzed by Mn(ii). Subsequently, Jwo *et al.*^10^ reported on a thorough study of the Mn(ii)-catalyzed BZ reaction in batch with MeMA as well as with other related acids of MA. The reaction was followed potentiometrically with a bromide ion selective electrode and periodic oscillations were obtained. In both of these studies using Mn(ii) as catalyst only simple periodic oscillations were obtained.

Recently, Frerichs *et al.*^11^ reported the surprising result that the closed BZ system containing BrO_3_^−^, MA, and Mn(ii) gave significant pH oscillations under a variety of conditions. The conclusion was that the pH oscillations were due primarily to the reversible formation of a manganese(iii) complex with bromomalonic acid. Because of the close similarity in their structures, it was decided to search for pH oscillations with the BZ reaction using MeMA in place of MA. Although significant pH oscillations were not found, a variety of fascinating complex oscillations, to our knowledge never reported before in this system, were observed.

Our goal in this study is to uncover the wide range of oscillation patterns exhibited by the Mn(ii)-catalyzed BZ reaction with MeMA as the organic substrate in a batch reactor. The oscillation patterns found include simple periodic oscillations, mixed mode oscillations (MMO), birhythmicity (BI), bursting (BR), quasiperiodicity (Q), and possible chaotic oscillations. We first describe the experimental procedure used, present the results in relation to how different conditions of reaction mixture composition, temperature, and stirring rate affect the oscillation patterns, then discuss observed oscillations in the context of a possible reaction mechanism for the BZ system, and finally provide a conclusion.

## Experimental section

Experiments were carried out in a glass reactor covered by a reactor cap and having a total solution volume of about 50 mL. The cap had a small opening covered by ParaFilm having a very small perforation. A Pt electrode and a combination pH electrode, each with a Ag/AgCl reference electrode, were inserted in the reactor cap. Both potential and pH were recorded concurrently using a Servogor 124 chart recorder. Stirring was done at various rates from 308 to 886 rpm using a Teflon-coated stir bar and either a Fisher Scientific or Heidolph Hei-Standard stirrer. Runs were carried out at room temperature (21 ± 1) °C, as well as at 25, 30, and 40 °C using a constant-temperature circulator. The reagents NaBrO_3_ (Fisher), MeMA (TCI), NaBr (Fisher) and MnSO_4_·H_2_O (Fisher) were ACS-certified. Ultrapure water was bubbled with N_2_ for about an hour before use.

Initially, the procedure for experiments was to add the solid reagents directly to the water in the reactor. Later, some runs were done using pre-dissolved NaBrO_3_. Eventually, the procedure involved pre-dissolving both NaBrO_3_ and MeMA before adding MnSO_4_·H_2_O and NaBr in that order. In most runs, H_2_SO_4_ was used and was added before the other reagents. A few experiments were done using NaOH, and some were carried out without either acid or base. Since the reaction vessel used was a CSTR reactor with closed ports, a few experiments were carried out at room temperature using a 100 mL beaker and comparable results were obtained.

## Results

**Fig. 1:**
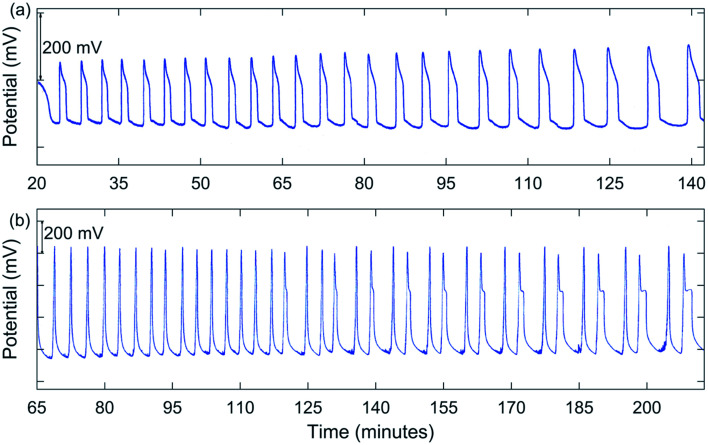
Measured Pt potential for the BZ-MeMA system in batch: (a) simple periodic oscillations at 25 °C. [NaBrO_3_]_0_ = 0.200 M; [MeMA]_0_ = 0.120 M; [NaBr]_0_ = 0.0200 M; [MnSO_4_]_0_ = 0.00600 M; [H_2_SO_4_]_0_ = 0.150 M.; (b) mixed mode oscillations [(3^1^(2^1^)_9_…] at room temperature. [NaBrO_3_]_0_ = 1.00 M; [MeMA]_0_ = 0.571 M; [NaBr]_0_ = 0.100 M; [MnSO_4_]_0_ = 0.0100 M; [H_2_SO_4_]_0_ = 0.010 M.

Early experiments involved using conditions somewhat comparable to those used by Jwo *et al.*, except [H_2_SO_4_]_0_ was lower in our study. Also, our experiments were carried out at room temperature with NaBrO_3_, MeMA, and MnSO_4_ being added as solids directly to an H_2_SO_4_ solution in the reactor. Not having had success in obtaining oscillations under these conditions, it was decided to include NaBr as a solid reagent. Large oscillations in potential were obtained using [NaBrO_3_]_0_ = 0.100–0.200 M, [MeMA]_0_ = 0.120 M, [NaBr]_0_ = 0.0100–0.0200 M, [MnSO_4_]_0_ = 0.00600 M, and [H_2_SO_4_]_0_ = 0.150–0.300 M. A typical result is shown in Fig. 1(a). Immediately after addition of all reactants the solution color was dark orange. During the induction period, the color went through the transition to lighter orange, yellow, and then nearly colorless before oscillations began. As each peak was formed, the solution color became pink to light tan.

In between oscillations, the solution again became nearly colorless, suggesting a transition from Mn(iii) to Mn(ii) ions. Oscillations were periodic, but not symmetric, as typically a “shoulder” appeared as the peak returned to the baseline. These oscillations continued for an extended period of time, sometimes for hours.

Since much higher [NaBrO_3_]_0_ and lower [H_2_SO_4_]_0_ were used in our investigation with MA, it was decided to follow the same approach in the present study with MeMA. With [NaBrO_3_]_0_ = 0.800 M, [MeMA]_0_ = 0.250–0.500 M, [NaBr]_0_ = 0.0800 M, [MnSO_4_]_0_ = 0.00600–0.00750 M, and [H_2_SO_4_]_0_ = 0.0150–0.0375 M, periodic oscillations occurred as in the earlier experiments, but in this case a brown color was observed during formation of a peak and an amber color in between peaks.

A significant change in the nature of the oscillations occurred when [NaBrO_3_]_0_ was increased to 1.00 M. Runs were carried out with [MeMA]_0_ = 0.400–1.25 M, [NaBr]_0_ = 0.100 M, [MnSO_4_]_0_ = 0.0100 M, and [H_2_SO_4_]_0_ = 0.0100 M. Under these conditions complex oscillations were observed for the first time. These were of the mixed mode types 2^1^ and 3^1^, using the notation *L*^*S*^ where *L* is the number of large-amplitude oscillations followed by *S* small-amplitude oscillations. One such result is shown in Fig. 1(b).

Subsequently, a series of runs was done with varying [MeMA]_0_, not only for 1.00 M NaBrO_3_, but also for 1.60 M and 2.00 M NaBrO_3_. An overall summary of the results is given in Table 1. Reactant concentrations used for NaBr, MnSO_4_, and H_2_SO_4_ are given in the table. A variety of mixed mode oscillations (MMO) was obtained, ranging from 2^1^ to 10^2^.

**Table:** Dependence of oscillations on [NaBrO_3_]_0_/[MeMA]_0_ ratio

Ratio	Cap time (min)	Ind. time (min)	Type of oscillations
**Initial concentrations: 1.00 M NaBrO**_**3**_ **; 0.100 M NaBr; 0.0100 M MnSO**_**4**_ **; 0.0100 M H**_**2**_ **SO**_**4**_ **; room temp.**			
2.50 : 1	19	45	(2^1^)_5_2^0^
2.00 : 1	17.5	47	Simple periodic oscillations
1.75 : 1	12	31	3^1^(2^1^)_9_…
1.50 : 1	9	23	(3^1^)_5_(2^1^)_3_(3^1^2^1^)_2_(2^1^)_2_2^0^
1.33 : 1	8	19	(2^1^)_11_…
1.00 : 1	5	15	(3^1^)_12_…
0.80 : 1	4.5	10.5	Simple periodic oscillations

**Initial concentrations: 1.60 M NaBrO**_**3**_ **; 0.160 M NaBr; 0.0100 M MnSO**_**4**_ **; 0.0188 M H**_**2**_ **SO**_**4**_ **; room temp.**			
4.44 : 1	37.5	56	(2^1^)_2_1^0^
4.20 : 1	35.4	53	(2^1^)_3_2^0^
4.00 : 1	28	42	(2^1^)_5_2^0^
3.60 : 1	25.5	38	(2^1^)_6_(1^1^)_5_1^0^
3.20 : 1	18.5	33	(2^1^)_14_2^0^
2.80 : 1	16.9	28.5	2^1^(3^1^)_3_(2^1^)_11_(2^2^)_2_2^*n*^
2.50 : 1	14.1	22	(2^1^)_13_2^*n*^
2.22 : 1	12.5	22	3^1^(2^1^)_11_2^2^2^3^2^*n*^
2.00 : 1	12.4	22.8	(2^1^)_16_(2^2^)_2_2^3^2^4^2^*n*^
1.75 : 1	11.5	18.5	3^1^2^1^(3^1^)_2_(2^1^)_2_(3^1^)_2_2^1^3^1^(2^1^)_4_3^1^(2^1^)_3_2^1^
1.50 : 1	8.4	12.4	(2^1^)_13_3^2^2^2^3^2^3^3^3^4^3^9^3^*n*^
1.25 : 1	7.2	12	(3^1^)_6_2^1^(3^1^)_3_3^2^(3^3^)_2_3^4^3^6^3^*n*^
1.00 : 1	5.6	9	Simple periodic oscillations

**Initial concentrations: 2.00 M NaBrO**_**3**_ **; 0.200 M NaBr; 0.0150 M MnSO**_**4**_ **; 0.0100 M H**_**2**_ **SO**_**4**_ **; room temp.**			
3.70 : 1	23.5	40	(3^1^)_2_3^0^
3.50 : 1	22	35	3^1^4^1^4^2^3^*n*^
3.20 : 1	18	33	(3^1^)_6_2^1^2^*n*^
2.80 : 1	14	22	2^2^4^1^(3^1^)_6_2^1^3^1^3^2^3^*n*^
2.50 : 1	12	19	(3^1^)_4_3^2^3^3^3^5^3^*n*^
2.25 : 1	12	19	(3^1^)_6_3^2^3^3^3^4^3^*n*^
2.00 : 1	9	14	(3^1^)_7_(3^2^)_2_3^3^3^4^3^7^3^*n*^
1.50 : 1	7.5	11.5	4^1^5^1^(4^1^)_9_…4^*n*^
1.25 : 1	5.2	8	(5^1^)_8_5^5^5^*n*^
1.12 : 1	4	6.8	7^1^6^1^6^2^(6^*n*^)_3_
1.00 : 1	4.4	6.8	10^2^9^3^8^*n*^
0.80 : 1	3.4	5.1	Shifting clusters of peaks

Perhaps the principal conclusions to be drawn from Table 1 are that the [NaBrO_3_]_0_/[MeMA]_0_ ratio is the main determinant of whether complex oscillations are obtained, and of what type. For runs done with 1.00 M NaBrO_3_, MMO were observed over a reactant ratio range of 2.50 : 1 to 1.00 : 1. With 1.60 M NaBrO_3_, MMO were found over a range of 4.44 : 1 to 1.25 : 1, and with 2.00 M NaBrO_3_ the reactant ratio range giving complex oscillations was 3.70 : 1 to 1.00 : 1. Also, the greater the initial concentration of NaBrO_3_ the more complex the oscillations tended to be. Further, for a given [NaBrO_3_]_0_ the complexity of oscillations generally increased with decreasing [NaBrO_3_]_0_/[MeMA]_0_ ratio (*i.e.*, with increasing [MeMA]_0_). Finally, for a given [NaBrO_3_]_0_ both the cap times and induction times tended to decrease with decreasing [NaBrO_3_]_0_/[MeMA]_0_ ratio. The “cap” refers to the broad maximum that generally occurs during the early part of the run with the transition from increasing to decreasing potential. This precedes a more gradual decrease in potential that leads to the onset of oscillations.

**Fig. 2:**
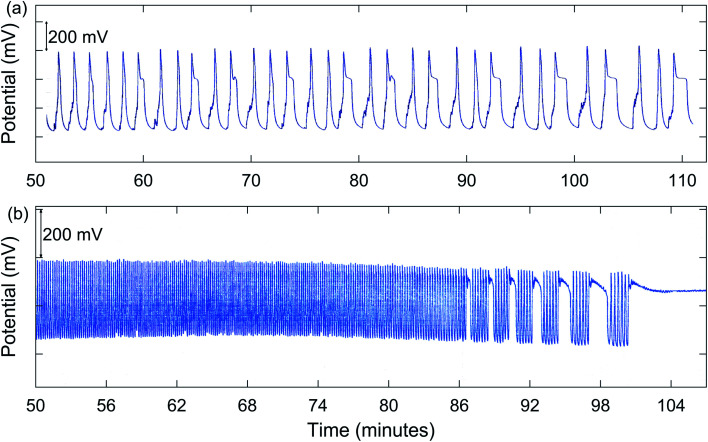
Measured Pt potential for the BZ-MeMA system in batch at room temperature: (a) chaotic oscillations [3^1^2^1^(3^1^)_2_(2^1^)_2_(3^1^)_2_2^1^3^1^…]. [NaBrO_3_]_0_ = 1.00 M; [MeMA]_0_ = 0.667 M; [NaBr]_0_ = 0.100 M; [MnSO_4_]_0_ = 0.0100 M; [H_2_SO_4_]_0_ = 0.010 M; (b) bursting oscillations [7^1^(6^1^)_5_]. [NaBrO_3_]_0_ = 2.00 M; [MeMA]_0_ = 1.79 M; [NaBr]_0_ = 0.200 M; [MnSO_4_]_0_ = 0.0150 M; [H_2_SO_4_]_0_ = 0.010 M.

In addition to simple MMO, other types of complex oscillations are suggested by the results summarized in Table 1. Numerous experiments demonstrated possible chaos. A good example is for 1.00 M NaBrO_3_ with a [NaBrO_3_]_0_/[MeMA]_0_ ratio of 1.50 : 1, as shown in Fig. 2(a). Also, bursting appears to be present for 2.00 M NaBrO_3_, especially at values of [NaBrO_3_]_0_/[MeMA]_0_ from 1.50 : 1 to 1.00 : 1. This phenomenon, which occurs when periods of quiescent behavior alternate with periods of relatively fast large-amplitude oscillations, is illustrated in Fig. 2(b).

Using the procedure described above, with [NaBrO_3_]_0_ = 1.60 M and [MeMA]_0_ = 0.500 M, the dependence of oscillations on temperature, [NaBr]_0_, [MnSO_4_]_0_, and presence of acid or base was investigated. The results are summarized in Table 2. Regarding the effect of temperature, similar MMO were observed at room temperature, at 20 °C, and at 25 °C, the main difference being the number of 2^1^ oscillations. Not surprisingly, the cap time, induction time, and period of oscillations all decreased with increasing temperature. As to the dependence of oscillations on [NaBr]_0_, good MMO were found with 0.160 M and 0.240 M NaBr, but the former had many more 2^1^ oscillations. Interestingly, both the cap time and induction time were reduced as [NaBr]_0_ was lowered. Also, similar MMO were obtained with 0.0100 M and 0.0120 M MnSO_4_, although the former gave significantly more 2^1^ oscillations. Regarding the impact of acid or base, good MMO were obtained with 0.0188 M H_2_SO_4_, with 0.010 M and 0.020 M NaOH, as well as with no acid or base added. One difference was that 2^1^ oscillations were found with H_2_SO_4_, whereas 3^1^ oscillations were the main type observed with NaOH or with no added acid or base.

Dependence of oscillations on temperature, [NaBr]_0_, [MnSO_4_]_0_, and [H_2_SO_4_]_0_ or [NaOH]_0_

**Table:** 

Initial Conditions: 1.60 M NaBrO_3_; 0.500 M MeMA; 0.160 M NaBr; 0.0100 M MnSO_4_; 0.01888 M H_2_SO_4_				
(°C) temperature	Cap time (min)	Ind. time (min)	Period (min)	Type of oscillations
Room temp.	18.5	33	5–6	(2^1^)_14_2^0^
20	20	34	8.5–12.4	(2^1^)_11_2^0^
25	14	25	5.4–7	(2^1^)_13_2^0^
30	9.6	14.8	1.8–3.2	(1^1^)_13_1^*n*^
40	3.5	5.2	1.8–3.2	(1^1^)_6_1^*n*^

**Table:** 

Initial conditions: 1.60 M NaBrO_3_; 0.500 M MeMA; 0.0100 M MnSO_4_; 0.0188 M H_2_SO_4_; room temp.				
[NaBr]_0_, M	Cap time (min)	Ind. time (min)	Period (min)	Type of oscillations
0.280	38.4	66.6	—	1^0^2^1^2_0_
0.240	31.4	50.6	6–7.2	1^1^(2^1^)_6_2^0^
0.160	18.5	33	5–6	(2^1^)_14_2^0^
0.0800	14	19.2	3.8–5.4	(2^2^)_2_(1^1^)_15_1^2^1^4^1^7^1^*n*^
0.0400	9	12.4	3–5.6	2^1^(1^1^)_18_(1^2^)_3_1^3^1^4^1^8^1^*n*^
None	22.2	24.8	3.6–6.2	(1^1^)_15_1^3^1^4^1^8^1^*n*^

**Table:** 

Initial conditions: 1.60 M NaBrO_3_; 0.500 M MeMA; 0.160 M NaBr; 0.0188 M H_2_SO_4_; room temp.				
[MnSO_4_]_0_, M	Cap time (min)	Ind. time (min)	Period (min)	Type of oscillations
0.0240	21.6	33.2	—	2^*n*^
0.0150	23.4	34.4	—	2^1^2^0^
0.0120	20.6	31.4	6	(2^1^)_7_2^4^2^*n*^
0.0100	18.5	33	5–6	(2^1^)_14_2^0^
0.00750	22	32.4	3.8–5.4	2^1^(1^1^)_15_1^0^
0.00500	24	36	5–7.2	2^1^

**Table:** 

Initial conditions: 1.60 M NaBrO_3_; 0.500 M MeMA; 0.160 M NaBr; 0.0100 M MnSO_4_; 25 °C				
[H_2_SO_4_]_0_ or [NaOH]_0_	Cap time (min)	Ind. time (min)	Period (min)	Type of oscillations
0.0500 M H_2_SO_4_	—	—	—	No oscillations
0.0350 M H_2_SO_4_	18	21.8	2.6–4.6	2^1^
0.0188 M H_2_SO_4_	14	25	5.4–7	(2^1^)_13_2^0^
No acid or base	11	38	9.8–10.6	(5^0^)_2_4^1^(3^1^)_6_2^1^2^0^
0.01 M NaOH	3.8	17.2	3–5.2	(6^0^)_2_4^0^(3^1^)_6_2^0^
0.02 M NaOH	8.2	50.6	12.5–14	(4^0^)_2_(4^1^)_2_(3^1^)_3_3^0^
0.03 M NaOH	18.5	23	3–5.3	2^1^

Because the dissolution of NaBrO_3_ in water is an endothermic process, and also to be sure the solid is completely dissolved before any reaction begins, it was decided to pre-dissolve NaBrO_3_ before adding it to the reactor. Also, the initial reaction of NaBrO_3_ with NaBr is quite exothermic. Thus, in order to eliminate any possible temperature effect, a constant-temperature circulator was used to maintain the reactor at 25 °C. A number of experiments were done using this procedure, including many runs with the same reactant concentrations as used previously. Although solid MeMA generally dissolved very readily after being added to the reactor, eventually it was decided to pre-dissolve this reactant as well. All subsequent experiments were done using the above method.

Since Ruoff and Schwitters^12^ had reported a stirring effect on the number and shape of oscillations in their study of the closed BZ system with MeMA and Ce(iv), it was thought important to investigate this effect also in the present system. Previously, all runs were carried out at the rather slow stirring rate of 308 rpm. To determine the effect of stirring rate, the present system was explored over a wide range from 308 to 886 rpm. Several previously used sets of concentrations were studied. The results are summarized in Table 3. It is clear that stirring rate can have a significant effect on the type of complex oscillatory behavior exhibited.

**Table:** Dependence of oscillations on stirring rate (25 °C)

**Code**: BI (birhythmicity); BR (bursting); MMO (mixed mode oscillations); Q (quasiperiodicity); P-1/2 (period-halving); NCO (no complex oscillations)	
Stir rate (rpm)	Behavior of system
**Initial concentrations: 1.00 M NaBrO**_**3**_ **; 0.667 M MeMA; 0.100 M NaBr; 0.0100 M MnSO**_**4**_ **; 0.0100 M H**_**2**_ **SO**_**4**_	
**379**	Lower state: simple periodic oscillations (2.5–11 mV)
BI,MMO	Higher state: (2^1^)_17_2^0^
**512**	Lower state: (3^1^)_4_4^1^3^1^(2^1^)_3_ (∼2 mV)
BI,MMO	Higher state: (2^1^)_4_2^0^
**635**	Lower state: simple periodic oscillations (33.5–37.5 mV)
BI	Higher state: simple periodic oscillations
**765**	Lower state: none
P-1/2	Higher state: (1^0^1^0^)_3_
**886**	Lower state: none
NCO	Higher state: simple periodic oscillations

**Initial concentrations: 1.60 M NaBrO**_**3**_ **; 0.500 M MeMA; 0.160 M NaBr; 0.0100 M MnSO**_**4**_ **; 0.0188 M H**_**2**_ **SO**_**4**_	
**0**	Lower state: none
MMO	Higher state: 1^0^(1^1^)_3_1^0^(1^1^)_5_1^0^(1^1^)_12_1^0^
**308**	Lower state: (2^1^)_4_ (19–20 mV)
BI,MMO	Higher state: (2^1^)_9_2^0^
**326**	Lower state: (3^1^)_5_ (5–10 mV)
BI,MMO	Higher state: (2^1^)_4_2^0^
**358**	Lower state: 3^1^ (11 mV)
BI,MMO	Higher state: (3^1^)_4_(2^1^)_3_2^0^
**400**	Lower state: (2^1^)_7_ (6–8 mV)
BI,MMO	Higher state: (2^1^)_5_2^0^
**512**	Lower state: 1^0^2^0^3^0^ (1–2 mV)
MMO	Higher state: (3^1^)_6_2 ^1^2^0^
**635**	Lower state: (3^0^)_3_ (1–2 mV)
MMO	Higher state: 3^1^5^1^(3^1^)_4_
**765**	Lower state: none
MM0	Higher state: (3^1^)_4_2^1^2^0^
**886**	Lower state: none
MMO	Higher state: 3^1^3^0^

**Initial concentrations: 2.00 M NaBrO**_**3**_ **; 1.33 M MeMA; 0.200 M NaBr; 0.0150 M MnSO**_**4**_ **; 0.0100 M H**_**2**_ **SO**_**4**_	
**308**	Lower state: 16 simple periodic oscillations (1–2 mV)
BI,Q,BR	Higher state: (5^1^)_2_3^1^(4^1^)_6_4^*n*^
**358**	Lower state: simple periodic oscillations (1–2 mV)
BI,Q,BR	Higher state: (4^1^)_6_(4^3^)_2_4^4^4^*n*^
**512**	Lower state: simple periodic oscillations (20–25 mV)
BI,Q,BR	Higher state: 5^1^9^1^6^1^5^1^(4^1^)_2_5^1^(4^1^)^2^4^0^
**635**	Lower state: simple periodic oscillations (1.5–2.5 mV)
BI,Q,BR	Higher state: 4^1^(5^1^)_2_4^1^(3^1^4^1^)_3_3^1^3^0^
**765**	Lower state: simple periodic oscillations (∼2 mV)
BI,Q,BR	Higher state: 10^1^8^1^5^1^(6^1^)_2_5^1^6^1^5^0^
**886**	Lower state: none
Q,BR	Higher state: (8^1^)_5_6^0^

**Initial concentrations: 2.00 M NaBrO**_**3**_ **; 1.60 M MeMA; 0.200 M NaBr; 0.0150 M MnSO**_**4**_ **; 0.0100 M H**_**2**_ **SO**_**4**_	
**358**	Lower state: none
Q,BR	Higher state: (6^1^)_2_(5^1^)_2_(4^1^)_4_4^0^
**512**	Lower state: none
Q,BR	Higher state: (5^1^)_7_5^0^
**635**	Lower state: none
MMO,Q,BR	Higher state: (2^1^)_2_9^1^(6^1^)_2_(5^1^)_3_4^0^
**765**	Lower state: none
Q,BR	Higher state: 7^1^(4^1^)_4_6^1^(4^1^)_4_4^0^
**886**	Lower state: none
MMO,Q,BR	Higher state: (2^1^)_2_8^1^5^1^4^1^(6^1^)_4_7^1^(5^1^)_2_4^0^

**Fig. 3:**
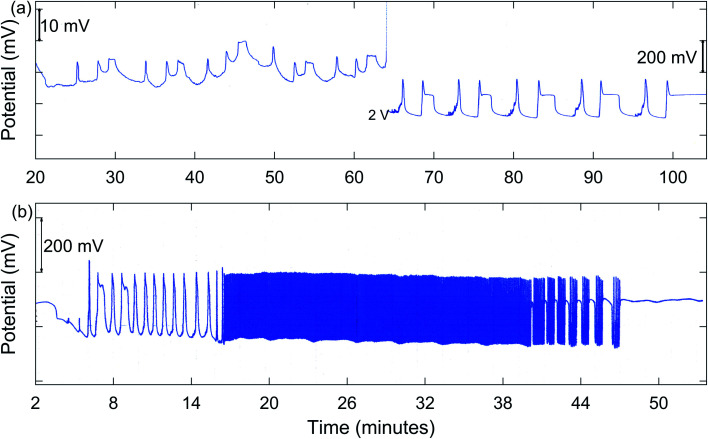
Measured Pt potential for the BZ-MeMA system in batch at 25 °C: (a) birhythmicity and mixed mode oscillations [3^1^)_5_(2^1^)_4_2^0^]. During transition from lower to higher state, potential full-scale setting was changed from 100 mV to 2 V. [NaBrO_3_]_0_ = 1.60 M; [MeMA]_0_ = 0.500 M; [NaBr]_0_ = 0.160 M; [MnSO_4_]_0_ = 0.0100 M; [H_2_SO_4_]_0_ = 0.0188 M. Stirring rate, 326 rpm; (b) mixed mode oscillations [(2^1^)_2_], simple periodic oscillations (9^0^), quasiperiodicity, and bursting oscillations [(6^1^)_2_(5^1^)_3_4^0^]. [NaBrO_3_]_0_ = 2.00 M; [MeMA]_0_ = 1.600 M; [NaBr]_0_ = 0.200 M; [MnSO_4_]_0_ = 0.0150 M; [H_2_SO_4_]_0_ = 0.0100 M. Stirring rate, 635 rpm.

In addition to MMO, birhythmicity (BI), bursting (BR), and quasiperiodicity (Q), along with possible chaos, were observed. An example of a run giving MMO and BI is shown in Fig. 3(a), and one showing a transition from MMO to Q and BR is given in Fig. 3(b). In the case of birhythmicity, a lower oscillatory state first appeared with peak amplitudes in the 1–40 mV range. Later in the run, there was a very large increase in potential to a higher oscillatory state and these peaks typically had amplitudes of 200–300 mV. Quasiperiodicity involves the appearance of two incommensurate frequencies of oscillations, one much lower than the other. With 1.00 M NaBrO_3_and 0.667 M MeMA, BI was observed at a stirring rate of 635 rpm or less, while both BI and MMO occurred at 379 and 512 rpm. Interestingly, at 765 rpm period-halving (P-1/2) took place, in which the system switched to a new behavior with half the period of the original oscillations. For the series of runs with 1.60 M NaBrO_3_ and 0.500 M MeMA, MMO were found at all stirring rates, but BI only at 400 rpm or lower.

With 2.00 M NaBrO_3_ and 1.33 M MeMA, BI, Q, and BR were all present except at the highest stir rate (886 rpm), which did not give BI. In the case of 2.00 M NaBrO_3_ and 1.60 M MeMA only the higher oscillatory state was present, with Q and BR being observed at all stirring rates but MMO only at 635 and 886 rpm. The results in Table 3, including experiments done with 2.00 M NaBrO_3_ and 1.60 M MeMA, suggest that Q and BR are more likely to be observed when [NaBrO_3_]_0_ is near the upper limit of concentrations giving complex oscillations, as long as [MeMA]_0_ is sufficiently high. It is to be noted from this table that possible chaos also is more likely to be found under these conditions.

## Discussion

Based on the similarity of the structures of MeMA and MA, it would be reasonable to expect the mechanisms of their reactions in the BZ system to be rather similar. A proposed model is given in Table 4. As with the model used for the BZ reaction with MA,^11^ we include the minimal bromate subsystem which generates Br_2_ and Mn^3+^. This is followed by the bromine–methylmalonic acid subsystem in which MeMA is converted to the enol, which then reacts with Br_2_ to produce bromomethylmalonic acid (BrMeMA). Finally, we include the manganese–bromomethylmalonic acid subsystem which features the reversible formation of a manganese(iii) complex with BrMeMA, followed by two-electron oxidation to methyltartronic acid (MeTA) and Mn^2+^. The fact that the solution color turns brown during the formation of a peak similarly to what was observed in the BZ reaction with MA lends support to the role of [Mn(iii)BrMeMA]^+^. For a typical run not showing birhythmicity, one can generally identify four rather distinct regions leading up to the appearance of oscillations. The reaction of BrO_3_^−^ with Br^−^ and H^+^ in step 1 of the proposed model takes place very rapidly, as evidenced by the nearly immediate appearance of a reddish-orange color apparently due to Br_2_ formation. Simultaneously, a very large increase in potential occurs. This is followed by a much slower reaction involving the reversible conversion of MeMA to the enol form (step 8). During this process a more gradual increase in potential takes place and generally a “cap” is formed. As this is occurring, the orange color turns yellow orange, indicating partial reaction of the newly formed enol with Br_2_. The next phase of the reaction corresponds to the somewhat faster reaction of enol with Br_2_ to form BrMeMA in step 9 of the model. There is a noticeable drop in potential during this step and the solution becomes yellow. Finally, there is another rather slow process (step 11) which is thought to be the reversible reaction of Mn^3+^ with BrMeMA to form the [Mn(iii)BrMeMA]^+^ complex. During this step, the potential drops further and the solution becomes light yellow. Eventually, oscillations begin, as indicated both by the increase in potential and the appearance of the brown color of the complex. During oscillations, the solution color then oscillates between amber and brown, likely due to the equilibrium between Mn^3+^ and [Mn(iii)BrMeMA]^+^ in step 11. Finally, oscillations stop when [Mn(iii)BrMeMA]^+^ is sufficiently depleted by conversion to MeTA and Mn^2+^ in step 12.

**Table:** Model for BZ batch oscillator with MeMA

**Minimal bromate subsystem**		
(1)	BrO_3_^−^ + Br^−^ + 2H^+^ ↔ HBrO_2_ + HOBr	*k*_1_ = 2.1 M^−3^ s^−1^; *k*_−1_ = 1 × 10^4^ M^−1^ s^−1^
(2)	HBrO_2_ + Br^−^ + H^+^ ↔ 2HOBr	*k*_2_ = 2 × 10^9^ M^−2^ s^−1^; *k*_−2_ = 5.0 × 10^−5^ M^−1^ s^−1^
(3)	HOBr + Br^−^ + H^+^ ↔ Br_2_ + H_2_O	*k*_3_ = 8 × 10^9^ M^−2^ s^−1^; *k*_−3_ = 110 s^−1^
(4)	BrO_3_^−^ + HBrO_2_ + H^+^ ↔ 2BrO_2_ + H_2_O	*k*_4_ = 1 × 10^4^ M^−2^ s^−1^; *k*_−4_ = 2.0 × 10^7^ M^−1^ s^−1^
(5)	Mn^2+^ + BrO_2_ + H^+^ ↔ Mn^3+^ + HBrO_2_	*k*_5_ = 1.8 × 10^5^ M^−2^ s^−1^; *k*_−5_ = 2.4 × 10^7^ M^−1^ s^−1^
(6)	Mn^3+^ + BrO_2_ + H_2_O ↔ Mn^2+^ + BrO_3_^−^ + 2H^+^	*k*_6_ = 35 M^−1^ s^−1^; *k*_−6_ = 1.3 × 10^−4^ M^−3^ s^−1^
(7)	2HBrO_2_ ↔ BrO_3_^−^ + HOBr + H^+^	*k*_7_ = 4 × 10^7^ M^−1^ s^−1^; *k*_−7_ = 2.1 × 10^−10^ M^−2^ s^−1^
**Bromine–methylmalonic acid subsystem**		
(8)	MeMA ↔ ENOL	*k*_8_ = 4.5 × 10^−4^ s^−1^; *k*_−8_ = 100 s^−1^
(9)	ENOL + Br_2_ → BrMeMA + Br^−^ + H^+^	*k*_9_ = 5 × 10^4^ M^−1^ s^−1^
(10)	MeMA + HOBr → BrMeMA + H_2_O	*k*_10_ = 80 M^−1^ s^−1^
**Manganese–bromomethylmalonic acid subsystem**		
(11)	Mn^3+^ + BrMeMA ↔ [Mn(iii)BrMeMA]^+^ + 2H^+^	*k*_11_ = 5 × 10^5^ M^−1^ s^−1^; *k*_−11_ = 1 × 10^5^ M^−2^ s^−1^
(12)	Mn^3+^ + [Mn(iii)BrMeMA]^+^ → MeTA + 2Mn^2+^ + Br^−^ + H^+^	*k*_12_ = 6 × 10^2^ M^−1^ s^−1^
**Abbreviations**		
MeMA ∼ CH_3_CH(COOH)_2_ ∼ methylmalonic acid		
ENOL ∼ (HOOC)C(CH_3_) = C(OH)_2_		
BrMeMA ∼ CH_3_BrC(COOH)_2_ ∼ bromomethylmalonic acid		
MeTA ∼ CH_3_COH(COOH)_2_ ∼ methyltartronic acid		

Computer simulations based on the model in Table 4 were carried out with Berkeley Madonna software using the Rosenbrock method for integrating stiff differential equations. The rate constants used for the minimal bromate subsystem are based on previously published values.^11^ Over many years, the kinetics of the enolization of MeMA as in step (8) has been studied by several investigators, including Furrow,^13^ Ruoff *et al.*,^9,12,14,15^ Williams and Graham,^16^ and Yoshimoto *et al.*^17^ Their results as summarized in Table 5 reflect a disparity of nearly an order of magnitude in *k*_8_. Data from the latter two references mentioned above allow an estimate of the value of *k*_−8_ for the reverse of the enolization reaction, but they do not agree. However, there is agreement that *k*_−8_ ≫ *k*_8_.

**Table:** Rate constant values for enolization of MeMA

*k*_8_ (s^−1^)	Ref.
1.63 × 10^−4^	13
5.56 × 10^−5^	12
5.7 × 10^−5^	14
4.87 × 10^−5^	9
5.2 × 10^−5^	15
3.96 × 10^−4^	16
4.4 × 10^−4^	17

The rate constant values for reactions in the bromine–methylmalonic acid and manganese–bromomethylmalonic acid subsystems were obtained by treating them as variable parameters in computer simulations using the model in Table 4.

Before attempting to reproduce the complex oscillations obtained in most of the experiments, we first tried to simulate the simpler periodic oscillations observed using lower concentrations of reactants as in Fig. 1(a). To determine the importance of [Mn(iii)BrMeMA]^+^, simulations were attempted without including the manganese–bromomethylmalonic acid subsystem (*i.e.*, letting *k*_11_ = *k*_−11_ = *k*_12_ = 0). Interestingly, it was not possible to produce oscillations under these conditions.

**Fig. 4:**
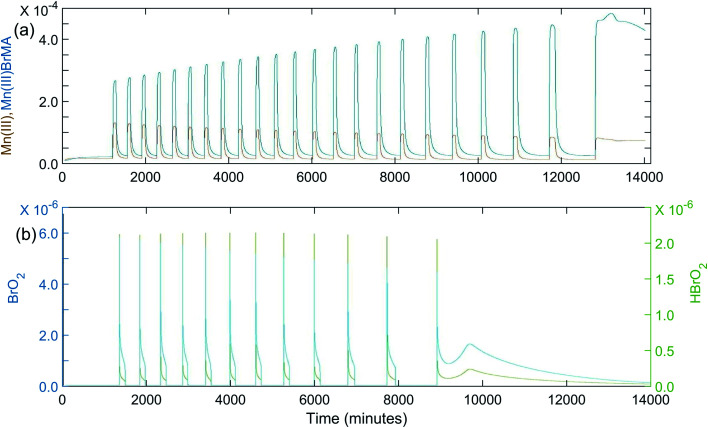
Calculated oscillations for the BZ-MeMA system in batch at 25 °C. [NaBrO_3_]_0_ = 0.200 M; [MeMA]_0_ = 0.120 M; [NaBr]_0_ = 0.0200 M; [MnSO_4_]_0_ = 0.00600 M; [H^+^]_0_ = 0.300 M. Time axis units are seconds: (a) [Mn^3+^]_0_ (brown) and [Mn(iii)BrMeMA^+^]_0_ (blue) using rate constants given in Table 4; (b) [BrO_2_]_0_ (blue) and [HBrO_2_]_0_ (green) using rate constants given in Table 4 except *k*_10_ = 40 M^−1^ s^−1^.

Once it was determined the manganese–bromomethylmalonic acid subsystem needed to be part of the model, it was found that the set of rate constants giving the best fit under the experimental conditions for Fig. 1(a) was: *k*_8_ = 4.5 × 10^−4^ s^−1^; *k*_−8_ = 100 s^−1^; *k*_9_ = 5 × 10^4^ M^−1^ s^−1^; *k*_10_ = 80 M^−1^ s^−1^; *k*_11_ = 5 × 10^5^ M^−1^ s^−1^; *k*_−11_ = 1 × 10^5^ M^−2^ s^−1^; *k*_12_ = 600 M^−1^ s^−1^. The calculated oscillations under these conditions are shown in Fig. 4(a). Note that these oscillations give good agreement with experiment in terms of the induction period, period of oscillations, and total number of peaks observed. Also, as the period increases toward the latter part of the experiment, the peaks begin to show a broadening or formation of a “shoulder,” as observed experimentally. This is especially evident in Fig. 4(b) where a *k*_10_ value of 40 M^−1^ s^−1^ is used in the plot of [BrO_2_] and [HBrO_2_] *vs.*time. This suggests that the intermediates, BrO_2_ and HBrO_2_, may play a significant role in determining the shape of the oscillatory peaks.

Although some of the rate constant values were treated as adjustable parameters in the simulations, nearly all of these values seem quite reasonable. For example, both the enolization rate and the rate of reaction of Br_2_ with MeMA have been shown experimentally to be significantly lower than for comparable reactions with MA. This is reflected in the smaller rate constant values used for steps (8) and (9) with MeMA. The only adjustable rate constant used in the simulations with a value larger than that for a similar reaction with MA is *k*_10_, the rate constant for reaction of MeMA with HOBr. While oscillations can be obtained with a *k*_10_ value comparable to that for reaction with MA (8.2 M^−1^ s^−1^), a significantly larger value is required to give the proper number of peaks with MeMA. Simulations show that, the larger the value of *k*_10_, the greater the number of oscillations formed. Also, the period of oscillations depends greatly on *k*_12_--the larger its value, the shorter the period. This seems reasonable, considering that *k*_12_ corresponds to the last step in the manganese–bromomethylmalonic acid subsystem.

Attempts to simulate the complex oscillations observed in this study, such as in Fig. 1(b) and 2, were unsuccessful. However, by using [H^+^]_0_ values larger than those used in our experiments (*e.g.*, 0.25–0.50 M) simple periodic oscillations could be obtained. This suggests that the model in Table 4 is not complete and that one or more steps producing H^+^ may be required in order to simulate the complex oscillations.

Ruoff *et al.*^18,19^ have studied the oxidation of MeMA by cerium(iv) and found that in this case about 70–80% of MeMA reacts rapidly to form acetic acid (HOAc) and CO_2_. Their proposed scheme suggests the oxidation of MeMA to MeMAOH at a moderate rate followed by the rapid conversion of MeMAOH to pyruvic acid (Pyr). Finally, Pyr is converted rapidly to HOAc. Assuming the same principal oxidation products are formed with the Mn(ii)-catalyzed system, it would seem reasonable to add steps (13) and (14) to the manganese–bromomalonic acid subsystem, thus giving: (12) Mn^3+^ + [Mn(iii)BrMeMA]^+^ → MeMAOH + 2Mn^2+^ + Br^−^ + H^+^ (slow)(13) MeMAOH + 2Mn^3+^ → Pyr + 2Mn^2+^ + CO_2_ + 2H^+^ (fast)(14) Pyr + 2Mn^3+^ + H_2_O → HOAc + 2Mn^2+^ + CO_2_ + 2H^+^ (fast)

The sum of steps (12), (13), and (14) would give an overall stoichiometry of:5Mn^3+^ + [Mn(iii)BrMeMA]^+^ + H_2_O → HOAc + 6Mn^2+^ + Br^−^ + 2CO_2_ + 5H^+^

However, using reasonable values for *k*_12_, *k*_13_, and *k*_14_ gave simulations very similar to those obtained without including steps (13) and (14).

Another possible reaction that could produce additional H^+^ is one suggested by Hanazaki *et al.*^20^ to partially account for pH oscillations with the bromate-sulfite system in a CSTR:3Mn^2+^ + BrO_3_^−^ + 3H_2_O → 3MnO(OH)^+^ + Br^−^ + 3H^+^

This reaction is thought to involve autocatalytic production of MnO(OH)^+^ with Mn(OH)^2+^ as an intermediate. Including the above reaction in our model again gave simulations similar to those obtained previously. If MnO(OH)^+^ were to play a significant role in the model, it possibly could oxidize MeMA according to the reaction:MnO(OH)^+^ + MeMA + H^+^ → Mn^2+^ + MeMAOH + H_2_O

However, the addition of this reaction likewise has no effect on the simulations. Apparently, MnO(OH)^+^ is not present to a sufficient extent in the present system which typically is in a pH range of 1–2.

## Conclusions

We have obtained both periodic and complex oscillations in the potential of a Pt electrode in batch with the Mn(ii)-catalyzed BZ reaction involving MeMA. Types of complex behavior observed include mixed mode oscillations (MMO), birhythmicity (BI), bursting (BR), quasiperiodicity (Q), and possible chaotic oscillations. Complex oscillations occurred only with rather high initial concentrations of NaBrO_3_ and MeMA. Systematic variations in the temperature, stirring rate, initial bromate/methylmalonic acid ratio, and initial concentrations of bromide, Mn(ii), and acid or base allowed us to elucidate oscillation patterns with respect to the reaction conditions. The [NaBrO_3_]_0_/[MeMA]_0_ ratio is the main factor determining whether complex oscillations are obtained, and of what type. Other factors found to affect the specific type of oscillations observed were temperature, [NaBr]_0_, [MnSO_4_]_0_, [H_2_SO_4_]_0_ or [NaOH]_0_, and stirring rate.

This is the first report of complex oscillations in the BZ reaction with MeMA in a stirred system. Because of the simplicity of the experimental procedure, the batch system lends itself to use in teaching laboratories. The procedure only involves dissolving three solid reagents in water (and possibly either H_2_SO_4_ or NaOH). Depending on conditions, various types of complex oscillations in potential and absorbance can be observed.

A model very similar to that for the Mn(ii)-catalyzed BZ reaction with MA has been proposed for the present system. The minimal bromate subsystem is the same as with other Mn(ii)-catalyzed BZ reactions. The bromine–methylmalonic acid subsystem involves previously reported reactions that parallel those with MA. A critical part of the mechanism involves the reversible formation of a manganese(iii) complex with BrMeMA, followed by two-electron oxidation to MeTA and Mn^2+^. In addition to the appearance of a brown color during oscillations, strong evidence for including the manganese–bromomethylmalonic acid subsystem is that periodic oscillations could be obtained by computer simulation only if [Mn(iii)BrMeMA]^+^ is included in the mechanism. (This suggests that the Mn(iii) complex may be formed in all Mn(ii)-catalyzed BZ reactions with MA or MeMA, but in some cases may not be present at a high enough concentration that it can be detected.) Although simulation of periodic oscillations with the proposed model was successful, this was not the case for complex oscillations. It is suggested that one or more steps involving formation of H^+^ may need to be added to the model in order to simulate complex oscillations. This could involve formation of further oxidation products, such as Pyr and/or HOAc.

It is interesting that bursting behavior similar to that described above was originally reported for biological systems. For example, bursting due to a changing membrane potential is common in various types of neurons, including pacemaker neurons. The latter have been shown to control rhythmic tasks such as breathing, locomotion, eating, and sleeping.^21^ Bursting also has been studied in various electrochemical systems, including H_2_O_2_ reduction on platinum, iron dissolution in sulfuric acid with halogen additives, and with dichromate ions coupled with graphite or zinc electrodes.^22^ In addition, the chlorine dioxide-iodide reaction in a system consisting of two coupled CSTRs (continuous-flow stir tank reactors) has been found to give neuron-like bursting behavior.^23^ The insight into the mechanisms that generate the observed oscillation patterns in the present work possibly could elucidate processes that lead to similar oscillation patterns in biological systems and other natural phenomena.

It is our hope that this study of the Mn(ii)-catalyzed BZ reaction using MeMA as the organic substrate presents new investigation opportunities in nonlinear chemical systems. For example, it would be informative to study this reaction in a flow system, especially to observe how the specific type of complex oscillations might vary with the reciprocal residence time *k*_0_.

## Conflicts of interest

There are no conflicts of interest to declare.

## Supplementary Material
